# Successful implantation of an EVOQUE-tricuspid valve replacement system in a patient with two right ventricular implantable cardioverter-defibrillator leads: a case report

**DOI:** 10.1093/ehjcr/ytaf066

**Published:** 2025-02-11

**Authors:** Florian Genske, Christoph Marquetand, Ingo Eitel, Christian Frerker, Tobias Schmidt

**Affiliations:** Medical Clinic II, University Hospital Schleswig-Holstein, University Heart Center Lübeck, Ratzeburger Allee 160, Lübeck 23538, Germany; DZHK (German Centre for Cardiovascular Research), Partner Site Hamburg/Kiel/Lübeck, Ratzeburger Allee 160, Lübeck, Germany; Medical Clinic II, University Hospital Schleswig-Holstein, University Heart Center Lübeck, Ratzeburger Allee 160, Lübeck 23538, Germany; DZHK (German Centre for Cardiovascular Research), Partner Site Hamburg/Kiel/Lübeck, Ratzeburger Allee 160, Lübeck, Germany; Medical Clinic II, University Hospital Schleswig-Holstein, University Heart Center Lübeck, Ratzeburger Allee 160, Lübeck 23538, Germany; DZHK (German Centre for Cardiovascular Research), Partner Site Hamburg/Kiel/Lübeck, Ratzeburger Allee 160, Lübeck, Germany; Medical Clinic II, University Hospital Schleswig-Holstein, University Heart Center Lübeck, Ratzeburger Allee 160, Lübeck 23538, Germany; DZHK (German Centre for Cardiovascular Research), Partner Site Hamburg/Kiel/Lübeck, Ratzeburger Allee 160, Lübeck, Germany; DZHK (German Centre for Cardiovascular Research), Partner Site Hamburg/Kiel/Lübeck, Ratzeburger Allee 160, Lübeck, Germany; Department of Cardiology, Asklepios Westklinikum Hamburg-Rissen, Suurheid 20, 22559 Hamburg, Germany

**Keywords:** Transcatheter tricuspid valve replacement, Tricuspid regurgitation, EVOQUE, Case report

## Abstract

**Background:**

Tricuspid regurgitation (TR) is a disease with significant morbidity and mortality rates. Besides surgery and transcatheter edge-to-edge repair (TEER), transcatheter tricuspid valve replacement has evolved as a possible treatment option in high-risk patients with an unfavourable anatomy for TEER.

**Case summary:**

We present a case of an 82-year-old patient with torrential TR due to an annulus dilation and subsequent central gap of >8 mm. Echocardiographic guiding was impeded by the presence of two permanent right ventricular implantable cardioverter-defibrillator (ICD) leads and previous surgical aortic valve implantation and mitral valve reconstruction. An EVOQUE prosthesis (Edwards Lifesciences; Irvine, USA) was successfully implanted without impairment of the ICD function or significant paravalvular leakage.

**Conclusion:**

Transcatheter tricuspid valve replacement with the EVOQUE-tricuspid valve replacement system is feasible even in patients with two right ventricular ICD leads and limited echocardiographic visibility.

**Learning points**

- The EVOQUE transcatheter tricuspid valve replacement (TTVR) system is a safe and feasible treatment option for patients with severe or higher-grade tricuspid valve regurgitation.
- The EVOQUE TTVR can be safely implanted, even in the presence of one or two right ventricular implantable cardioverter-defibrillator leads.

## Introduction

Tricuspid regurgitation (TR) is a common valvular heart disease with significant morbidity and mortality rates.^1–3^ Current European guidelines recommend optimal medical therapy and list surgical treatment of severe isolated TR in symptomatic patients as a Class I recommendation.^4^ Tricuspid valve transcatheter edge-to-edge repair (T-TEER) has evolved as a safe and effective treatment option over the last years.^5,6^ However, T-TEER is limited in patients with massive or torrential TR and/or large coaptation gaps.^5,7^ Leaflet impingement due to previously implanted pacemaker or cardioverter-defibrillator (ICD) may also impair its feasibility.^8^

## Summary figure

**Figure 1:**
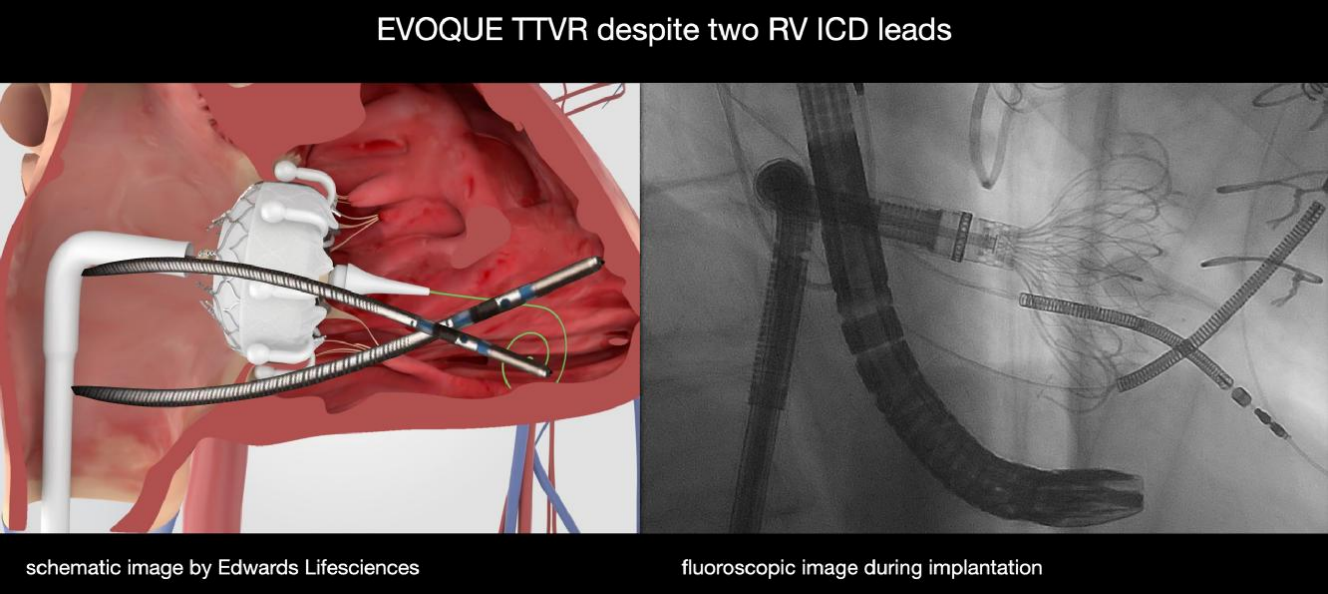
Central illustration shows a side-by-side view of the EVOQUE prosthesis (Edwards Lifesciences) as a schematic image and as a fluoroscopic image during implantation.

Transcatheter tricuspid valve replacement (TTVR), such as the EVOQUE prosthesis (Edwards Lifesciences; Irvine, USA) is a promising new treatment option. Good procedural safety and favourable clinical outcomes of TTVR at 1 year have recently been reported.^2,9^ Both, Kodali *et al*.^9^ and Webb *et al*.^2^ report a patient population of which one third had right ventricle (RV) leads prior to TTVR and showed that it is save to perform TTVR—even in patients with pre-existing RV leads. We report the first case of a patient who underwent successful TTVR with the EVOQUE prosthesis despite having two ICD leads (*Figure 1*).

## Case presentation

**Figure 2:**
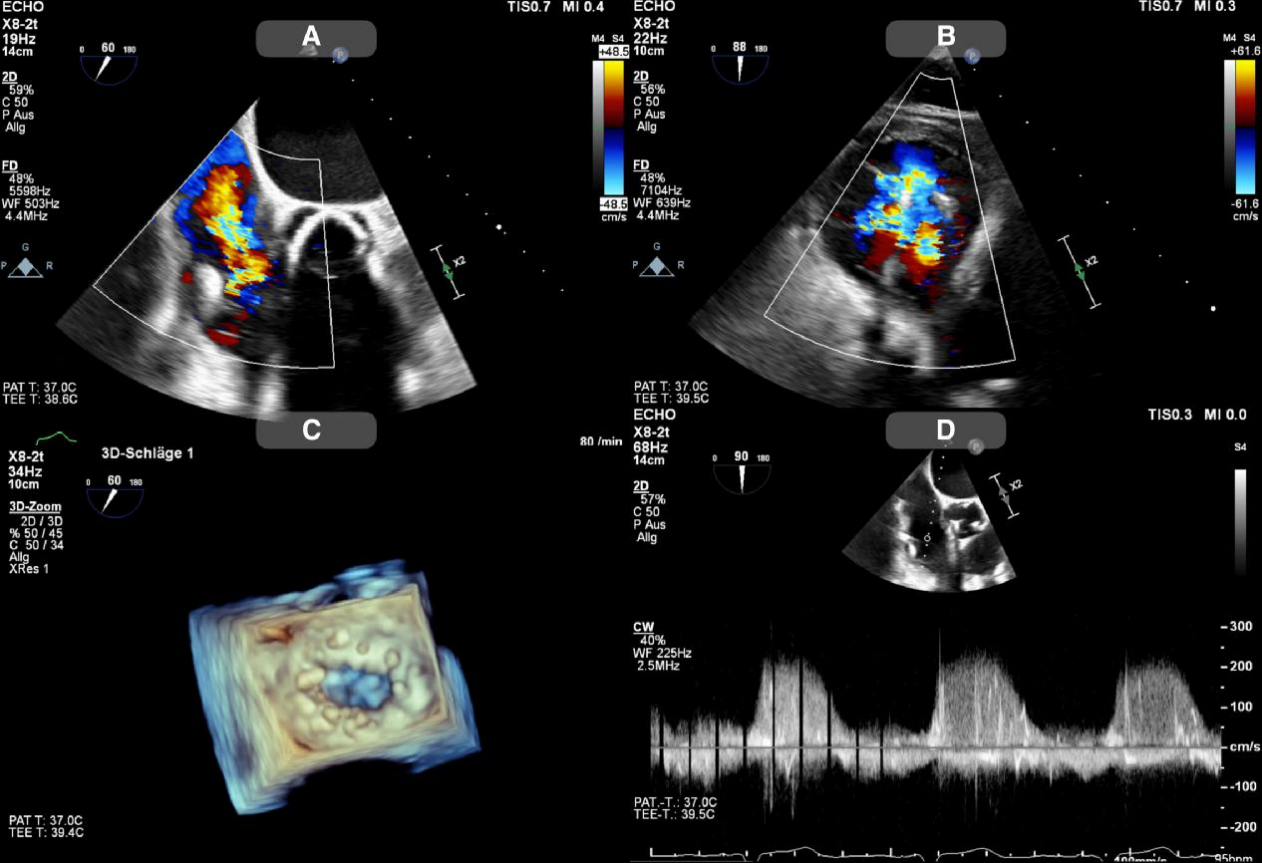
Pre-procedural echocardiographic assessment shows pre-procedural echocardiographic images of the tricuspid regurgitation. (*A* and *B*) Colour Doppler images of the torrential tricuspid valve insufficiency. (*C*) 3D reconstruction of the large central coaptation gap. (*D*) Tricuspid regurgitation velocity within the normal range, thereby indicating no elevated pulmonary pressure.

An 82-year-old patient presented at our centre with decompensated heart failure, New York Heart Association (NYHA) Class III. Transoesophageal echocardiography (TEE) after decongestion revealed torrential TR with a large central coaptation gap of >8 mm (see *Figure 2*). Lead impingement, lead-associated TR and pulmonary hypertension were excluded. The further medical history of the patient included left heart failure with improved left ventricular ejection fraction (currently ∼54%) based on dilated cardiomyopathy and permanent atrial fibrillation. Right ventricle function was preserved but the right ventricle was severely enlarged. The patient had previously been equipped with an implantable cardiac resynchronization therapy defibrillator. During a replacement the RV lead could not be retrieved, thus a second lead was implanted. Furthermore, the patient had previously undergone cardiac surgery with surgical aortic valve replacement (SAVR) and mitral valve reconstruction.

The case was discussed in the interdisciplinary heart team and was regarded as unsuitable for surgical therapy because of the high surgical risk (log Euro-score: 38.38%; Society of Thoracic Surgeons [STS]-score: 14.8%, tri-score: 4/12). Interventional therapy by TEER was regarded unsuitable because of the exceptionally large central gap and the lack of suitable echocardiographic windows. The case was screened for TTVR and judged as borderline suitable. The discussion was held in accordance with the current guidelines on valvular heart diseases, where it is stated that transcatheter tricuspid valve implantation may be considered at experienced heart valve centres in symptomatic, inoperable, anatomically eligible patients in whom symptomatic or prognostic improvement can be expected.^4^ After an extensive discussion within the interventional team, device specialists from the company and echocardiography specialists, we concluded that the risk of damaging the ICD lead was low. Paravalvular leakage (PVL) was to be expected but to an acceptable extent. Especially the lack of optimal echocardiographic views was discussed intensively since multiplane reconstruction was not feasible. A pre-procedural computer tomography was performed to assess the tricuspid valve annulus and the venous vessels for access. Sizing of the device showed a good oversizing (of 10% diastolic tricuspid annulus) and the largest EVOQUE prosthesis (52 mm) was chosen for implantation.

### Intervention

**Figure 3:**
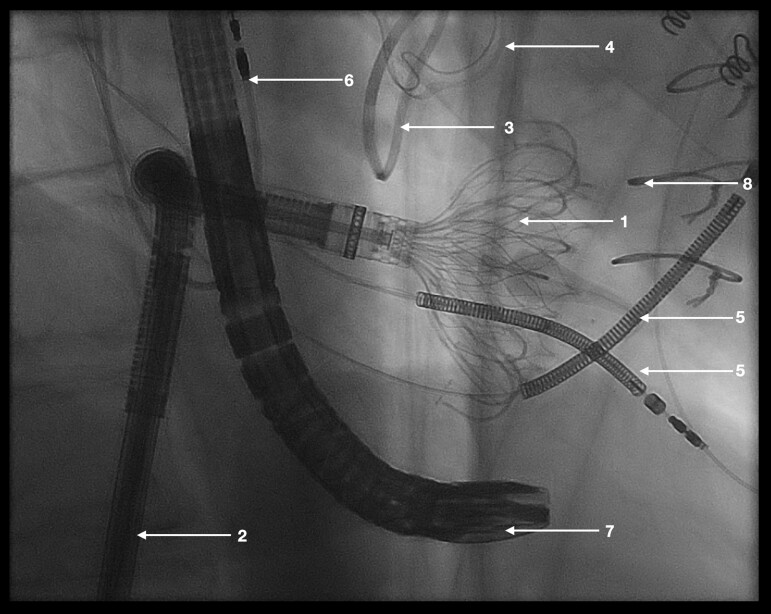
Fluoroscopy during implantation of the EVOQUE prosthesis shows a fluoroscopic image of the implantation process. 1—partially deployed EVOQUE prosthesis; 2—EVOQUE delivery system; 3—Carpentier–Edwards Physio II annuloplasty ring; 4–23 mm Carpentier–Edwards Magna Ease-Bioprosthesis; 5—right ventricular implantable cardioverter-defibrillator leads; 6—right atrial pacemaker lead; 7—transoesophageal echocardiography probe; 8—sternal cerclage.

**Figure 4:**
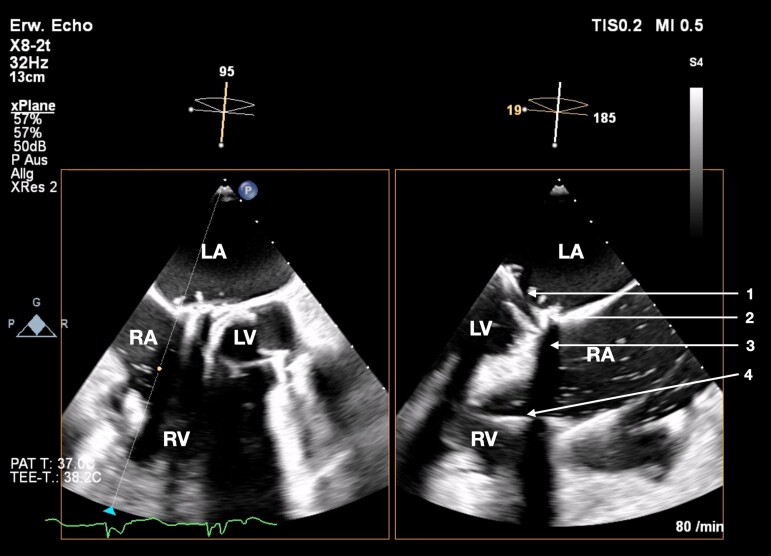
Transoesophageal echocardiography prior to EVOQUE implantation shows the transoesophageal echocardiography prior to implantation. Left: mid- to deep-oesophageal right ventricle inflow–outflow view; right: corresponding biplane view/inverted four-chamber view. The acoustic shadow from the aortic valve bioprosthesis and mitral valve reconstruction impairs the view. 1—mitral valve annuloplasty; 2—aortic valve bioprosthesis; 3—acoustic shadow; 4—implantable cardioverter-defibrillator lead (LA, left atrium; RA, right atrium; LV, left ventricle; RV, right ventricle).

**Figure 5:**
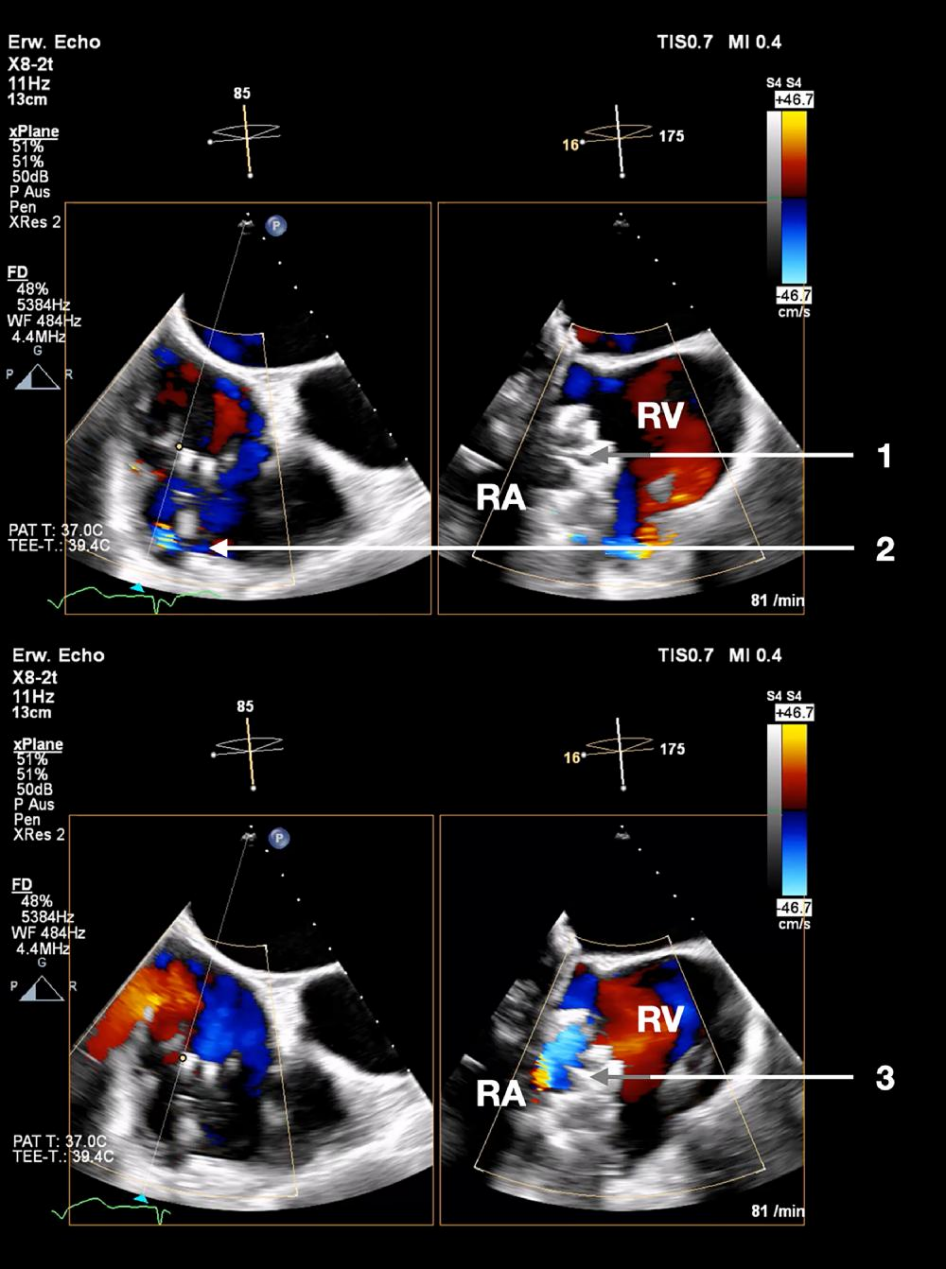
Post-procedural transoesophageal echocardiography showing the EVOQUE prosthesis shows a post-procedural transoesophageal echocardiographic colour Doppler image of the EVOQUE prosthesis. Arrow 1 shows the prosthesis during systole. No valvular regurgitation can be seen. Arrow 2 shows minimal PVL around the implantable cardioverter-defibrillator lead. Arrow 3 shows the prosthesis during diastole and the inflow jet through the prosthesis into the right ventricle (RA, right atrium; RV, right ventricle).

The procedure was carried out under general anaesthesia. Percutaneous vascular access was obtained through the right femoral vein. After dilation of the venous access site up to 38 French and advancement of an Agilis sheath (Abbott Cardiovascular; Chicago, USA) into the right atrium, followed by a Safari wire XS (Boston Scientific; Marlborough, USA), which was placed in the right ventricular apex, a 52 mm EVOQUE prosthesis was advanced into its position in the tricuspid valve annulus. A capsule gap was created to evaluate the depth of the prosthesis in the right ventricle. At this step, in the procedure, a point of no return was reached and the final decision to implant the prosthesis despite the challenging anatomy and the severely impeded echocardiographic view (standard echocardiographic views could not be utilized because of prior surgery with SAVR and mitral valve replacement, see *Figures 3* and *4*) was made after another careful discussion with all team members. The echocardiographic guidance was performed using mid- and deep-oesophageal as well as transgastric views in biplane 2D and 3D-multiplanar reconstruction. For each valve leaflet and anchor, the best individual acoustic windows were sought. After positioning of the device within the tricuspid valve annulus, the ventricular anchors, which grasp the tricuspid leaflets, were released. After the final release of the prosthesis no valvular regurgitation and only minimal PVL around the ICD-coils could be seen (TR trace, see *Figure 5*). The venous access site was closed with the two Prostyle systems (Abbott Cardiovascular; Chicago, USA) and a final skin suture. Due to the challenging anatomy of the patient and the limited echocardiographic views, finding the optimal echocardiographic windows for each step of the implantation was relatively time consuming. Therefore, the overall procedure time was 205 min.

Post-procedural transthoracic echocardiography showed an overall good result. Paravalvular leakage was hardly detectable, mean transvalvular gradient was 3 mmHg. Mild regurgitation (vena contracta < 3 mm) of the previously reconstructed mitral valve could be seen. The ICD leads could be seen jailed between the prosthesis and the RV wall. Post-procedural interrogation of the ICD revealed normal device function with normal impedances, pacing thresholds, and regular sensing.

The patient left our hospital 4 days after the procedure in good overall condition. A clinical and echocardiographic follow-up 3 months later showed an improvement in the patient’s overall condition (NYHA Class II, no peripheral oedema), no progress of the mean gradient and still no TR.

## Discussion

Regarding the significant morbidity and mortality rates of TR and the unfavourable outcomes of isolated tricuspid valve surgery or medical therapy alone, the demand for treatment alternatives rises.^10,11^ The EVOQUE prosthesis offers a safe and feasible alternative.^2,9^

Recent studies have shown that TTVR is such an option, even in patients with transtricuspid leads.^2,9^ Pacemaker lead displacement or lead malfunction has been described during and after TTVR in a study by Anderson *et al*.^12^ However, no differences in the competing outcomes were observed between patients with and without pacing leads. Kodali *et al*.^9^ and Webb *et al*.^2^ both describe a patient population of which about one-third had RV pacemaker or ICD leads prior to TTVR and both do not report failure of the leads or unfavourable functional outcomes after TTVR.

The EVOQUE prosthesis uses anchor-like structures to secure its position within the annulus. These anchors do not exert significant pressure on the annulus or the sub-valvular structures. Therefore, direct damage to pacemaker or ICD leads is unlikely, especially since the sensing and pacing electrode of the leads are at their tip but the prosthesis is implanted at a considerable distance from the tip. However, dislocation leading to an exit block may occur if the lead comes under tension. Paravalvular leakage is minimized by the intra-annular sealing skirt, even for patients with RV leads. In the above presented case, this is of particular interest, as our patient had not one, but two Implantable cardioverter-defibrillator (ICD) leads. ICD leads have a substantially larger diameter than pacemaker leads with standard pacemaker leads having a 4.1 French diameter and ICD leads having a diameter between 6.8 and 8.6 French.^13^

The use of intracardiac echocardiography (ICE) has been previously described as an adjunct to TEE to aid TTVR,^14^ but official recommendations are lacking. In the above-described case ICE was not utilized as it was not available at our centre. In our case the patient underwent successful TTVR despite the severe difficulties in echocardiographic imaging caused by the significant amount of alien material.

Our case underlines that TTVR with the EVOQUE system can be safely performed, even in patients with pre-existing transtricuspid leads. Future iterations in device design and careful simulation of frame/anchor interaction with pre-existing pacing leads will endorse the EVOQUE system as a new therapeutic option for patients with severe TR.

## Data Availability

The data underlying this article cannot be shared publicly to preserve patient anonymity. The anonymized data will be shared on reasonable request to the corresponding author.

## Lead author biography

Florian Genske is a cardiologist in training at the university hospital in Luebeck with a special interest in transcatheter valve interventions.

## Notes

**Consent**: Informed consent was obtained from the patient for publication of this case report according to the Committee of Publication Ethics (COPE) guidelines.

**Funding:** None declared.
